# How the experiences of implementation support recipients contribute to implementation outcomes

**DOI:** 10.3389/frhs.2024.1323807

**Published:** 2024-06-19

**Authors:** Allison J. Metz, Todd M. Jensen, Jenny L. Afkinich, Mackensie E. Disbennett, Amanda B. Farley

**Affiliations:** School of Social Work, University of North Carolina at Chapel Hill, Chapel Hill, NC, United States

**Keywords:** implementation practice, implementation support, mechanisms of change, implementation science, implementation outcomes

## Abstract

**Introduction:**

There is a growing body of literature on the activities and competencies of implementation support practitioners (ISPs) and the outcomes of engaging ISPs to support implementation efforts. However, there remains limited knowledge about the experiences of implementation support recipients who engage with ISPs and how these experiences shape the trajectory of implementation and contribute to implementation outcomes. This study aimed to extend the research on ISPs by describing the experiences of professionals who received implementation support and inform our understanding of the mechanisms by which ISPs produce behavior change and contribute to implementation outcomes.

**Methods:**

Thirteen individuals with roles in supporting implementation efforts at a private foundation participated in semi-structured interviews. Data were analyzed using *qualitative narrative analysis* and *episode profile analysis* approaches. Iterative diagramming was used to visualize the pathway of experiences of implementation support recipients evidenced by the interview data.

**Results:**

The majority of recipients described how positive experiences and trusting relationships with ISPs increased acceptance of implementation science throughout the foundation and increased the perception of implementation science as both an appropriate and feasible approach for strengthening the impact of foundation strategies. As perceptions of appropriateness and feasibility increased, recipients of implementation support described increasing knowledge and application of implementation science in their funding engagements and internal foundation strategies. Finally, recipients reported that the application of implementation science across the foundation led to sustained implementation capacity and better outcomes.

**Discussion:**

The experiences of implementation support recipients described in this paper provide a source for further understanding the mechanisms of change for delivering effective implementation support leading to better implementation quality. Insights from these experiences can enhance our understanding for building implementation capacity and the rationales for evolving approaches that emphasize the dynamic, emotional, and highly relational nature of supporting others to use evidence in practice.

## Introduction

In utilizing implementation support, individuals and organizations often collaborate with implementation support practitioners (ISPs), whose explicit role is to support the implementation, sustainment, and scaling of evidence-informed practices, programs, and policies to improve outcomes for focus populations. Recent work has conceptualized and defined this role (1, 2), including an articulation of how ISPs can be located outside the service systems they support or operate from *within* a service system when those systems have internal work units specifically designed to support innovation, implementation, improvement and/or scaling efforts. Implementation support is often delivered through partnerships between professionals residing inside and outside public service systems. Recent work has also described a shared set of values and competencies (2) and knowledge and attitudes (3) that inform and guide the work of ISPs to successfully use implementation strategies to support sustainable change in service systems.

ISPs have been identified as a potential strategy for building implementation capacity in service systems (2). Some studies have described the role of professionals in providing implementation support as a promising approach for achieving and sustaining quality implementation (4, 5). Recent research has demonstrated a link between the implementation support delivered by ISPs and the achievement of implementation goals (6, 7) and higher rates of use of evidence-based practices (8). Albers and colleagues present a conceptual model connecting the resources provided by ISPs, including their knowledge, skills, and attitudes, the context where implementation support is delivered, and changes in capability, motivation, and opportunity of implementation partners resulting in implementation outcomes (9).

There is a growing body of literature on the activities and competencies of ISPs and the outcomes of engaging ISPs to support implementation efforts. However, findings from a systematic integrative review on mechanisms through which ISPs produce behavior change in recipients of implementation support found few empirical studies that enhanced our understanding of how the efforts of ISPs contributed to behavior change and implementation outcomes (9). Based on the dearth of studies focused on this topic, Albers and colleagues (10) highlight the complexity of capturing the mechanisms of change for implementation support and hypothesize that change in the attitudes and behaviors of those receiving implementation support is dependent on factors at multiple levels of the service system; however, the ISPs use of specific skills stood out as a variable that triggered responses from implementation support recipients. This same systematic review (9) foregrounded the role of relational responses for implementation support recipients, describing how the use of specific skills by ISPs can produce responses related to trust and high-quality relationships among those receiving implementation support.

Given the complexity of capturing the mechanisms by which implementation support produces implementation outcomes, further investigations are needed into ISPs' collaboration with recipients of implementation support. Specifically, more implementation studies are needed on how ISPs make choices about the strategies and approaches they use based on recipients' attitudes towards implementation support and other contextual factors of the implementation setting (11, 12). Previous research has demonstrated that ISPs spend as much time brokering connections, addressing power differentials, and building relationships as they do on more technical work such as conducting improvement cycles (2), suggesting that ISPs view the quality and mutuality of their relationships with recipients of implementation support as critical for implementation success. In a recent study, Metz, Jensen, Farley and Boaz (13) document how ISPs with extensive experience supporting the use of evidence-based practice in service systems have moved from push models of implementation support (i.e., one-directional, didactic models of support) to exchange models focused on co-creation and relationship-based modes of support.

Formalized implementation support has also been described in theoretical and conceptual contributions to implementation support, including the early description of external implementation support in the Interactive Systems Framework (14) taxonomies of implementation strategies (15), and research on implementation facilitation and the role of ISPs (1, 2). Aldridge and colleagues (16) recently presented a conceptual model on the mechanisms of change for external implementation support grounded in social cognitive theory.

Theory-driven, empirical work on the role of ISPs in producing implementation outcomes, though, has focused solely on the perspective of ISPs, rather than the perspective of those receiving implementation support. In order to better understand the mechanisms by which ISPs produce change in the behavior of implementation support recipients, we need to unearth and describe the experiences of those receiving support and how those experiences contribute to implementation outcomes. This study seeks to extend the current research on the role of ISPs by describing the experiences of professionals who received implementation support and how these experiences shape the outcomes achieved through their engagement with ISPs.

## Methods

### Study setting and participants

The current study focused on individuals with active roles in a private foundation supporting implementation of internal and external initiatives to improve health, educational, economic, and social outcomes for children and young people in the United States. The foundation has almost 200 staff and funds over 700 grantees. The sample reflected all units within the foundation that were engaged in implementation support, with at least two individuals invited to participate from each unit. Participants were invited to ensure positional diversity within the foundation including senior leadership, managers, and support roles (e.g., leader and support staff from one unit). These individuals from each unit formed implementation teams for the engagement period. Participants were also selected based on whether their implementation support was “high engagement” (12 + months) or “limited engagement” (6–12 months). Additional data related to demographic information for each participant were not available for secondary data analysis.

All engagements consisted of monthly meetings with implementation teams across units to assess implementation support needs and monitor implementation progress on a range of initiatives related to improving outcomes for children, young people, and families engaged in public systems (e.g., child welfare, juvenile justice) and participating in educational and career opportunities (e.g., apprenticeships, financial coaching). Each engagement began with a request from a unit for implementation support related to an internal initiative or on behalf of an external partner or grantee. A series of meetings were then scheduled to more clearly define the implementation problem using qualitative methods, followed by a co-learning process designed to identify implementation goals, implementation support strategies, and indicators of implementation progress. Specific deliverables would also be discussed, which ranged from operationalization of core components for replication, recommended scaling approaches, and stage-based implementation guidance.

While implementation strategies for each engagement were tailored for specific needs and service contexts, all engagements were steeped in a co-learning approach and included monthly in person and virtual implementation support. The implementation support team consisted of five ISPs from a university-based implementation science center, each with a minimum of 5 years' experience providing implementation support. All ISPs have extensive knowledge of implementation science theories, models, and frameworks. For each implementation support engagement, two ISPs served as co-leads, and other members of the ISP team provided additional support as needed. Implementation coaching was provided to implementation teams and leaders between monthly meetings; and products, tools, and resources were developed to support the use of evidence-based implementation strategies. ISPs did not have prior relationships with implementation support recipients.

Between November 2020 and February 2021, recipients of implementation support were invited to be interviewed about their implementation support experiences. The electronic invitation letter asked 13 recipients to participate in a 60-min virtual interview where they would be asked to provide feedback on the implementation support they received. The core aim of the interviews was to guide efforts to improve the future delivery of implementation support and identify key lessons learned. The final sample was comprised of the 13 individuals who supported implementation efforts within and outside the foundation and were engaged in implementation support activities, each of whom was interviewed once. Fifty percent of respondents were White. Specific sociodemographic characteristics are not available for the full sample of interviewees, in large part because the data were initially collected for internal purposes only. Although the original aim of the data collection effort was to highlight learnings from recent implementation support engagements to inform internal processes, both on the part of those providing implementation support and those receiving it, as we engaged with the data it became clear that some of the insights we were gleaning could add value to the implementation science literature. Thus, following internal review of key interview findings by both providers and recipients of implementation support, we submitted an application (study #: 22-2516) to our university's Office of Human Research Ethics, whereby we proposed use of fully de-identified data for further analysis. Following review, the Office of Human Research Ethics determined that the submitted request did not constitute human subjects research as defined under federal regulations [45 CFR 46.102 (e or l) and 21 CFR 56.102(c)(e)(l)] and did not require further Institutional Review Board approval.

### Data collection procedures

Data were collected via in-depth, semi-structured interviews. Interviews were 60–75 min in duration. The Zoom web-conferencing platform was used to engage with participants and record interviews. Interview prompts were developed by the research team in alignment with the following core foci: (a) the nature of participants' relationship with the ISP team; (b) how they have experienced their implementation support related to communication and partnership; (c) the strengths and limitations of implementation support; (d) ways in which their implementation support needs were acknowledged and addressed; and (e) whether and how implementation support contributed to changes in motivation and capacity to use implementation science individually, within their unit, and across the foundation. Participants received the interview prompts in advance. One member of the research team with implementation science expertise and extensive training in quantitative and qualitative methods led the interviews, and another member of the research team attended the interviews to observe and engage in general notetaking. The lead interviewer (second author) was a member of the research team who had not delivered implementation support. Audio recordings from each interview were transcribed verbatim in preparation for analysis.

### Data analysis

To begin we applied the *sort and sift, think and shift* approach as outlined by Maietta and colleagues (17), which encourages an initial process of data familiarization to inform the selection of specific analytic techniques suitable for the aims of a particular study. Following the research team's familiarization with the transcript data, we leveraged a narrative analytic framework and developed episode profiles whereby rich summary information was compiled for each individual interview (the focal unit of analysis), followed by a synthesis and thematic aggregation of all episode profiles (17, 18).

Episode profiles for each interview were developed via two core stages of analysis, both of which were accompanied by detailed memoing and note-taking. In the first stage, two members of the research team engaged the data while reflecting on (a) two-to-three key takeaways from the interview, (b) general insights related to participants' lived experiences while receiving implementation support, and (c) general connections between participants' responses and extant implementation support competencies (and associated skills) as outlined in recent literature (2). In stage two, the same two members of the research team re-engaged the data while reflecting on (a) specific mechanisms for change (i.e., process or event through which an implementation strategy operated to affect desired implementation outcomes), (b) contextual factors that helped or hindered the use of implementation science, and (c) implementation outcomes linked to the receipt of implementation support.

Following each of the two stages of analysis, the full research team met to reflect on emergent findings and triangulate general interpretations of study data to garner coherence and consensus across research team members. Diagramming was then used to develop a visual aggregation of the (a) experiences of implementation support recipients and (b) how those experiences shaped implementation outcomes from recipients' perspective (19). The diagram then became the focal analytic object by which the research team could refine and finalize a representative aggregation of participant experiences.

## Results

All implementation support recipients described ISPs as using the full array of skills related to the ISP competencies defined by Metz and colleagues (2) including skills related to co-creation and engagement (e.g., co-design, tailoring support), ongoing improvement (e.g., facilitation, improvement cycles), and sustaining change (e.g., building relationships, developing teams). All recipients also described the implementation support they received as wide ranging, noting that ISPs “*provided structure, management, and communication*” (R4) for all implementation activities. As noted earlier, implementation support typically consisted of monthly in-person implementation team meetings with planning and debriefing meetings taking place virtually. ISPs conducted outreach, collected data, provided implementation coaching, and developed tools and resources to support implementation weekly or biweekly.

**Figure 1:**
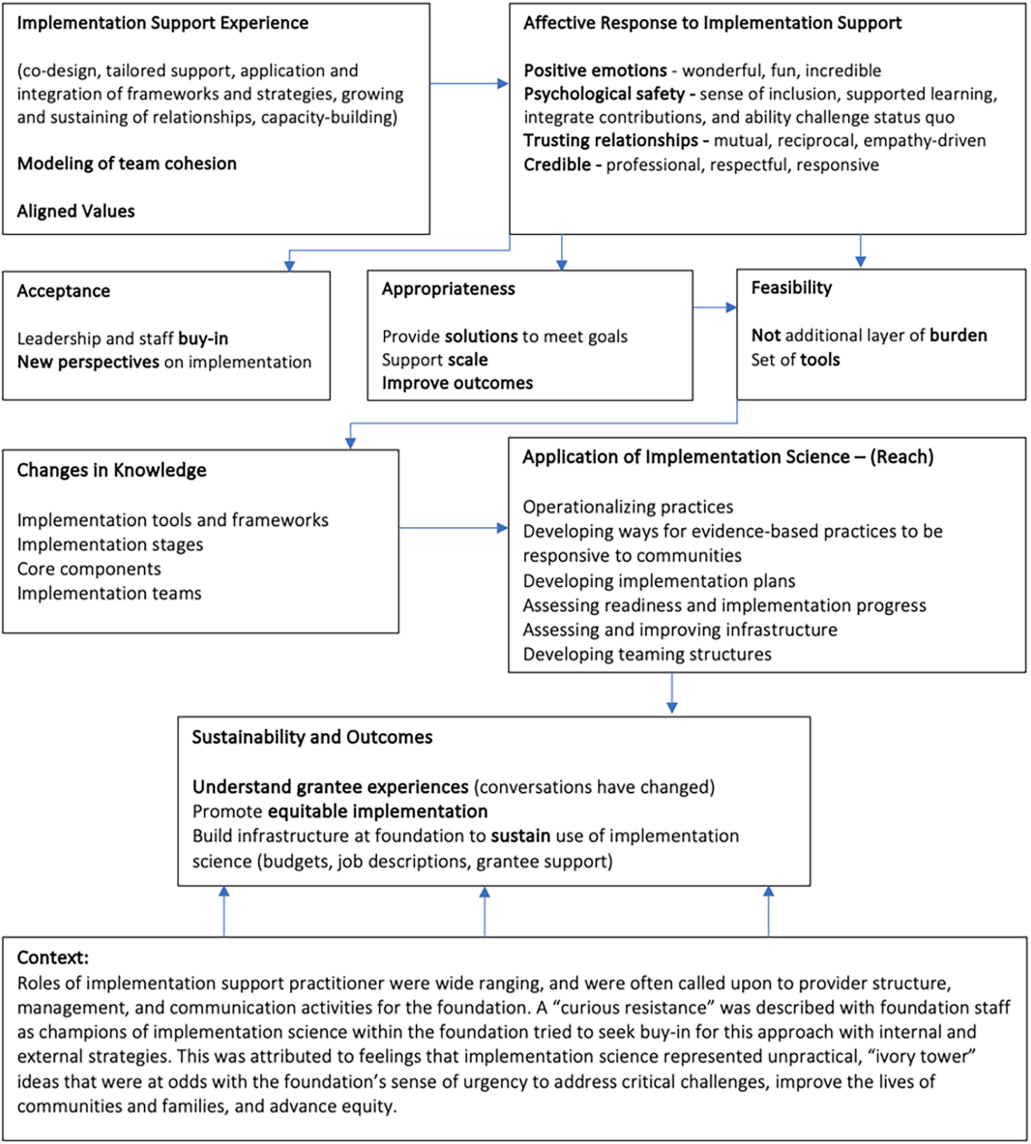
Experiences and outcomes pathway for recipients of implementation support.

Figure 1 visually depicts the experience and outcomes pathway for recipients of implementation support as articulated by recipients. The majority of recipients described how positive experiences and trusting relationships with ISPs increased acceptance of implementation science throughout the foundation and increased the perception of implementation science as both an appropriate and feasible approach for strengthening the impact of foundation strategies with focus populations and communities. As perceptions of appropriateness and feasibility increased, recipients of implementation support described being open to learning more about implementation science (increasing knowledge) and applying what they learned in their funding engagements and internal foundation strategies. Finally, recipients reported that the application of implementation science across the foundation led to sustained implementation capacity and better outcomes. Results did not differ based on the length of the implementation support engagement.

### Affective response to implementation support

Recipients emphasized their relational responses to implementation support in the context of an organizational culture that did not readily embrace the use of implementation science. Relational responses emerged as positive emotional responses to implementation support, perceived psychological safety on implementation teams, a commitment to trusting relationships between ISPs and recipients of implementation support, and respect for the credibility brought by ISPs with expertise in implementation science (e.g., knowledge of and experience using implementation theories, models, frameworks, and strategies).

All recipients described a positive affective response to engaging with the ISP team, noting that ISPs demonstrated “*curiosity, respect, and openness*” (R2). Recipients shared their perceptions of ISPs as “*flexible and authentic*” (R8) and committed to “*reciprocal relationships and partnerships*” (R6) with all recipients of implementation support. Recipients explained how crucial these relationships were to addressing potential resistance to implementation science, noting that previous research partnerships within the foundation had been experienced negatively by foundation staff. One recipient described the implementation support partnership as a different experience, “*I know this word gets used a lot, but I would say this is an authentic partnership*” (R11).

Recipients also described how ISPs contributed to a psychologically safe environment for implementation activities. Psychological safety refers to a shared belief held by members of a team that the team is safe for interpersonal risk taking (20). Recipients shared that in their work with ISPs they felt safe in a number of ways (21) including safe to be included, safe to learn, and safe to contribute, highlighting that “*ISPs demonstrated that the relationship [with foundation staff] was a mutual learning opportunity*” (R5).

Recipients also reported feeling safe to challenge the status quo due to the teaming structure and facilitation approach developed by the ISPs, reporting “*the interlocking teaming structure helps make sure there is continuity of information as well as different levels of authority that need to be involved in order to have something be supported completely [at every level]*” (R2).

Recipients described the ISPs as credible, noting the “*rigor and carefulness*” (R4) with which the implementation support team approached their work, while demonstrating a “*willingness to have honest conversations about what it will require for implementation efforts to be successful*” (R4). Credible work was done in the context of relationships with recipients noting that ISPs possessed “*great humility*” (R8) in the work and made it clear from the outset they were committed to an “*authentic partnership*” (R6). Recipients contrasted the “*humble and co-creative*” (R8) approach of the ISPs with other research-practice partnerships they had experienced that felt less reciprocal.

### Acceptability, appropriateness, and feasibility of using implementation science

Recipients described how the positive affective responses triggered by support from ISPs contributed to increases in the acceptability of using implementation science by foundation staff, as well as helped to increase the perceived appropriateness and feasibility of using implementation science strategies to support execution of the foundation's internal and external initiatives. For example, a recipient who shared that ISPs were “*professional, collaborative, and authentic*” (R11) also noted changes in the acceptability of implementation science as foundation staff demonstrated an “*increased awareness of the consideration of all steps needed in implementation before outcomes research and evaluation can enter the work*” (R11). Multiple recipients also commented on gaining new perspectives as implementation support shifted their sense of buy-in for implementation science. For example, one recipient noted “*the delivery of implementation support increased the understanding of its value across the organization*” (R11). Recipients also commented on the alignment of values that ISPs displayed with the foundation's approach to partnerships, including not entering the space as an expert, demonstrating curiosity, and frequently sharing what they were learning from implementation support recipients during the course of the implementation engagement.

Recipients described significant shifts in the perceived appropriateness of implementation science as an approach to help the foundation meet its strategic goals, support scaling of initiatives, and improve outcomes. For example, one participant within the foundation described their initial reaction to implementation science as “*What is this [implementation science]? How does it work?*” *What's it supposed to do?* “*How does it connect to our work?*” (R9). This same recipient explained how ISPs utilized established relationships to strengthen the contextual fit of implementation science with the foundation's day-to-day activities.

*The magic was when we started. We knew enough about their work. They knew enough about our work. We could go back and forth and figure out what framework, tool, or approach from implementation science would best fit the foundation's work and the goals of our leadership team (R9).*

Another recipient noted the connection between perceived appropriateness and feasibility for integrating implementation science into their day-to-day work.

*So, how do we do that [*“*replicate and scale…in a way that is as effective as possible*”*] … that doesn't feel like we're adding an extra layer of burden…I think implementation science has been valuable in that it's provided us with a set of tools that's allowing us to do our work better and that's allowing our partners to do their work better (R6).*

Additionally, recipients described the ISPs' focus on responsiveness and tailoring implementation strategies as critical for changing perceptions of implementation science as an “ivory tower” approach to something that is both practical and feasible to use. “*They [ISPs] are actively interested in understanding where we are and making sure that they are able to right-size their support for our goals*” (R6). Recipients commented on the importance of ISPs' ability to be “*nimble*” (R7) in their delivery of implementation support. “*They [ISPs] are more interested, just as we are, in result…It's not about their [ISPs] agenda, it's about our agenda and what they're trying to help us achieve*” (R9).

Another recipient described the adaptability of the ISPs noting “*another skill that is present with the [ISP] team is high adaptability…with attention to* ‘*what is going on now?*’ *and* ‘*how will this change what we are going to do?*’” (R11).

### Changes in knowledge and application of implementation science

As implementation support recipients began to see implementation science as both appropriate and feasible to use to help the foundation achieve its goals, recipients reported an openness to learning more about implementation science, leading to a deeper understanding of implementation science frameworks and principles and the ability to apply to these frameworks in their own work. For example, an implementation support recipient described learning about the importance of teams and communication to support implementation efforts.

*A team model versus a solo model of implementation support has several perceived benefits including versatility, depth and breadth of knowledge and expertise, cohesion, and continuity. Teams meet regularly, discuss strategy together, and share responsibility for the body of work (R2).*

Recipients shared that ISPs were able to build staff's knowledge on foundational implementation science concepts (e.g., implementation stages, core components) and implementation frameworks [e.g., Consolidated Framework for Implementation Research (22]) while also translating theories and frameworks into usable tools and practical resources that could become embedded in the foundation's way of work moving forward. One recipient emphasized that the ISPs used specific skills (e.g., relationship building, active listening) that aided in the translation of implementation science to real world application, noting:

*They [ISPs] were really good listeners, and they had the ability to both translate and tweak [implementation frameworks]. So, I don't know if that has more to do with the ISPs themselves, than the tools they had, but I think their ability to do that made the relationship [with the foundation] work (R12).*

Application of implementation science was wide-ranging with recipients of implementation science describing the following uses: (1) tailoring and conducting infrastructure assessments to guide improvements; (2) identifying core components to build fidelity criteria; (3) developing and convening implementation teams; (4) identifying stage-based benchmarks to assess implementation progress and guide implementation planning; (5) further operationalizing service models that demonstrate progress to support replication efforts.

Changes in knowledge coincided with application of implementation science. One recipient described how the ISPs helped to build the currency of implementation science over time, noting that implementation support recipients now understand “*there should be a bridge between evidence-building activities and the actual [implementation] support in grant-making provided to communities*” (R4).

### Sustainability and outcomes

Implementation support recipients described how implementation science has become embedded in the foundation's work.

*It is really the crux of how anything gets done and, so I feel as though I have learned an enormous amount about implementation research and implementation practice, and I see them as critical to making sure that we get the kinds of results we want for kids, families, and communities in everything that we do (R2).*

Recipients noted that foundation staff were making use of implementation action guides (i.e., brief topical guides describing equitable implementation best practices) and implementation tools (e.g., stage-based planning, processes for using data for improvement), developing implementation teams, and conducting various implementation assessments to determine and strengthen implementation readiness. Recipients shared that ISPs helped the foundation to “*shape and define*” (R7) what is meant by implementation science so that individual units were better equipped to communicate about the importance of implementation with grantees and communities.

Recipients reported that foundation budgets demonstrated internal support for the use of implementation science as individual foundation staff began to include notes on evidence-building and implementation in foundation budget requests. One recipient who is a foundation leader shared:

*I saw two things in almost every single programmatic budget justification document—evidence and implementation. I think that's a direct result of the work that we've done over the last two or three years…they [foundation staff] seem to know how to use it [implementation science] (R7).*

One recipient used the analogy of “*widening concentric circles*” (R4) to describe how implementation science developed a footprint within the foundation.

*Some people were very interested in the work, some people were skeptics, and some people were completely disinterested. Little by little as implementation support was positioned on new projects and introduced to more people at the foundation, these low-stakes interactions helped the resistance to melt away. People began to see those delivering implementation support as credible and sought their involvement on more projects (R4).*

Another recipient noted that one metric of sustainability for the use of implementation science at the foundation was the decision to apply implementation science to a new, foundation-wide initiative, which indicated that implementation science would be used more broadly by foundation staff in the future.

### Limitations and challenges to implementation support

Recipients of implementation support noted a “curious resistance” throughout the private foundation for the use of implementation science. This resistance was attributed to perceptions that implementation science represented unpractical, “ivory tower” ideas. One recipient explained, “*There is a natural inclination to keep moving [at the foundation] and forego creating learning opportunities. It is important to have a board that has bought into slowing down enough so you can implement well*” (R9). Another recipient noted the tension in systems

*between great ideas and the time it takes to use an implementation science framework to really implement it well. There is a sense of urgency for the problems we are trying to address and the slowing down can feel like missed opportunities (R9).*

However, all implementation support recipients also described a positive affective response to receiving implementation support that contributed to shifts in perception around slowing down to implement well. Recipients provided rich detail on how implementation support provided by the ISPs evoked such positive emotions, and subsequently how those positive emotions contributed to changing the attitudes and behavior of those at the foundation engaged in implementation efforts.

*Implementation science really tells you it is actually slowing down to speed up. And we might say those words, but we don’t always live by those words* *(R9).*

## Discussion

Findings from this study suggest that the affective responses of those receiving implementation support contribute to implementation outcomes. Moreover, this study provides emerging evidence that ISPs can play an important role in triggering the positive emotions that are needed for recipients to invest in implementation, even when those investments are counter to an organizational culture that values speed and immediate results over implementation quality. Results did not differ based on the length of the implementation support engagement, which underscores the importance of early interactions between ISPs and implementation support recipients and whether recipients experience those interactions positively.

Recipients of implementation support in the current study demonstrated developmental steps towards integrating and sustaining the use of evidence-based implementation strategies in their work and facilitating the achievement of key implementation outcomes for the foundation. All recipients emphasized their positive emotional responses to implementation support and described how this changed their perceptions of the feasibility and appropriateness of using implementation science in their work, and generally increased their buy-in for implementation science. Once recipients were bought in to implementation science, they invested in growing their knowledge, applying implementation best practices, and finding ways to sustain the use of implementation science in the foundation's internal strategy development and external grant-making activities.

Previous studies have described the complex and reciprocal mechanisms of change unfolding in the interactions between ISPs and those who receive implementation support (16). Conceptual models of implementation support are typically described from the perspective of those delivering implementation support (i.e., experts in implementation science). This study attempted to unpack these mechanisms of change through the perspectives of those *receiving* implementation support. Understanding what motivates recipients of implementation support to be open to learning about implementation science and integrating evidence-based implementation strategies into their implementation efforts is critical for unpacking the mechanisms of change for building sustainable implementation capacity in organizations, systems, and communities.

This study is particularly timely as the “secondary” research-to-practice gap has received increasing attention in the field of implementation science (23, 24). Observations in the field (2, 25, 26) have pointed to a growing disconnect between implementation research and implementation practice. A major part of this disconnect is a lack of “practical implementation science,” described by Meyers and colleagues (27) as the “user-friendly translation of implementation science results” (p. 4). This translation typically consists of the development and use of practical tools and resources to support implementation, as well as an implementation support system that delivers these tools and resources—in this case, the team of ISPs that actively supported implementation efforts through interactive capacity-building strategies.

Implementation researchers hypothesize that installing trust between ISPs and recipients of implementation support, as well as among implementation partners, leads to meaningful and relevant learning by implementation support recipients, which in turn motivates recipients to use implementation science to achieve better outcomes (9). Metz et al. (28) expand on this hypothesis by using relational cohesion theory to explain how the affective responses of those receiving implementation support is a critical contributor to trust-building. Relational cohesion theory emphasizes how relationships that emerge from positive affective experiences are valuable in and of themselves and contribute to the trusting relationships needed to increase motivation, commitment, and resilience during implementation efforts.

Implementation support provided by ISPs can only succeed if the conditions under which ISPs are working are supportive of their role and create the necessary space for implementation support to be delivered and received (9, 13). This study took place in a context that was not readily accepting of implementation science—and in some cases, actively resistant to implementation science. The conditions under which ISPs worked (e.g., early expectations of quick timeframes and minimal commitments to using implementation science) were not conducive to implementation science being integrated into day-to-day practices of the foundation. This context, though, is not uncommon for ISPs, and many ISPs find that part of their role involves actively building buy-in for implementation science as part of their practice. In a survey of ISPs conducted by Metz and colleagues (2), ISPs described how their role is often limited by organizational factors including the lack of a learning culture of the implementing site and limited absorptive capacity of the site, or “the ability of stakeholders and organizations to recognize value of new knowledge and seek sources of support for implementing a new practice” (p. 13). Therefore, understanding how positive affective responses from implementation support recipients may reduce resistance and build buy-in for implementation science is important for the development and testing of implementation strategies that trigger positive emotion among implementation partners and subsequently lead to meaningful learning and reflection on implementation best practices and motivation to engage in high quality implementation.

The experiences of implementation support recipients described in this paper provide an additional source for further understanding the mechanisms of change for delivering effective implementation support that leads to better implementation quality. Insights from the experiences of implementation support recipients can enhance our understanding for building implementation capacity and the rationales for evolving approaches that emphasize the dynamic, emotional, and highly relational nature of supporting others to use evidence in practice (9, 29).

### Limitations and future research

This study is limited by the single foundation setting for implementation support. Future research could explore the experiences of implementation support recipients in other settings including federal, state, and local public agencies, hospitals and clinics, community-based nonprofits, and higher education. These studies would allow for the identification of common experiences and context-specific experiences of implementation support recipients. Another limitation of this study is that implementation support was provided by a team of ISPs from a single university-based implementation science center who shared a set of competencies, values, and approaches for delivering implementation support. Future studies can assess whether and how the specific skills and approaches of ISPs contribute to variable experiences of those receiving implementation support, and consequently different implementation outcomes. Finally, this study involved secondary data analysis of interview data gathered to improve implementation support activities. Future studies that are designed at the outset to assess the experiences of implementation support recipients and how those experiences shape implementation outcomes can use more targeted methods and measures to better understand the complex dynamics of implementation support and how they are tethered to emotional responses, trust, and relationships between ISPs and recipients.

## Conclusion

As we seek to build implementation capacity in service systems, it is important that our research designs encompass the perspectives of those receiving implementation support, namely leaders, managers, and staff who are accountable for evidence use with the goal of improving population outcomes. This study begins to fill a gap in the literature related to the mechanisms of change for building implementation capacity based on the experiences of implementation support recipients. Disciplines other than implementation science such as clinical psychology, social work, and anthropology would point to the critical role of the human experience in shaping how recipients make sense of and use implementation science to achieve better outcomes. Results from this study offer emerging evidence that emotional responses to implementation support can serve as lever for increased acceptability of implementation science in service systems. Focusing on the complex dynamics of implementation support—and the interactions between ISPs and recipients—can help us identify and tailor implementation strategies that strengthen the relationships needed to build implementation capacity.

## Data Availability

The data analyzed in this study is subject to the following licenses/restrictions: the dataset is not available for public use. Requests to access these datasets should be directed to Allison J. Metz, allison.metz@unc.edu.
